# Weighing as a stand-alone intervention does not reduce excessive gestational weight gain compared to routine antenatal care: a systematic review and meta-analysis of randomised controlled trials

**DOI:** 10.1186/s12884-016-1207-2

**Published:** 2017-01-17

**Authors:** Shanna M. Fealy, Rachael M. Taylor, Maralyn Foureur, John Attia, Lyn Ebert, Alessandra Bisquera, Alexis J. Hure

**Affiliations:** 1 School of Nursing & Midwifery University of Newcastle, Port Macquarie Campus, PO Box 210, Port Macquarie, 2444 NSW Australia; 2 Faculty of Health & Medicine School of Medicine & Public Health, University of Newcastle, Callaghan, NSW Australia; 3 Maternity Care Services, The Port Macquarie Base Hospital, Port Macquarie, NSW Australia; 4 Mothers and Babies Research Centre, University of Newcastle, University Drive, Callaghan, 2308 NSW Australia; 5 Hunter Medical Research Institute, Newcastle, NSW Australia; 6 Centre for Midwifery, Child and Family Health, Faculty of Health, University of Technology Sydney, PO Box 123, Broadway, Ultimo, NSW 2007 Australia; 7 Centre for Clinical Epidemiology and Biostatistics, School of Medicine & Public Health, University of Newcastle, Callaghan, NSW Australia; 8 Division of Medicine, John Hunter Hospital, Newcastle, NSW Australia

**Keywords:** Antenatal care, Gestation, Pregnancy, Weighing, Weight gain

## Abstract

**Background:**

Excessive gestational weight gain is associated with short and long-term adverse maternal and infant health outcomes, independent of pre-pregnancy body mass index. Weighing pregnant women as a stand-alone intervention during antenatal visits is suggested to reduce pregnancy weight gain. In the absence of effective interventions to reduce excessive gestational gain within the real world setting, this study aims to test if routine weighing as a stand-alone intervention can reduce total pregnancy weight gain and, in particular, excessive gestational weight gain.

**Methods:**

A systematic review and meta–analysis of randomised controlled trials (RCTs) was conducted between November 2014 and January 2016, and reported using the Preferred Reporting Items for Systematic Reviews and Meta-Analyses. Seven databases were searched. A priori eligibility criteria were applied to published literature by at least two independent reviewers. Studies considered methodologically rigorous, as per the Academy of Nutrition and Dietetics Quality Criteria Checklist for Primary Research, were included. Meta-analysis was conducted using fixed-effects models.

**Results:**

A total of 5223 (non-duplicated) records were screened, resulting in two RCTs that were pooled for meta-analysis (*n* = 1068 randomised participants; *n* = 538 intervention, *n* = 534 control). No difference in total weight gain per week was observed between intervention and control groups (weighted mean difference (WMD) -0.00 kg/week, 95% confidence interval (CI) -0.03 to 0.02). There was also no reduction in excessive gestational weight gain between intervention and control, according to pre-pregnancy body mass index (BMI). However, total weight gain was lower in underweight women (*n* = 23, BMI <18.5 kg/m^2^) in the intervention compared to control group (−0.12 kg/week, 95% CI −0.23 to −0.01). No significant differences were observed for other pregnancy, birth and infant outcomes.

**Conclusion:**

Weighing as a stand-alone intervention is not worse nor better at reducing excessive gestational weight gain than routine antenatal care.

**Electronic supplementary material:**

The online version of this article (doi:10.1186/s12884-016-1207-2) contains supplementary material, which is available to authorized users.

## Background

Obesity has dramatic effects on reproductive health with complications during pregnancy and at birth all the more prevalent in those carrying excess weight [1]. Globally obesity is more prevalent than undernutrition [2]. The World Health Organisation (WHO) estimates that over 1.9 billion adults (≥18 years) are overweight and 600 million obese [3]. In Australia, 63% of adult women (≥18 years) are reported to have a body mass index (BMI) in the overweight (25.0–29.9 kg/m^2^) or obese (≥30.0 kg/m^2^) categories [4]. For women who gave birth in Australia, the most recent Mothers and Babies report (2013) shows that one-fifth (19%) of pregnant woman were classified as obese at the beginning of pregnancy with one quarter (24%) overweight [5].

The risks of entering pregnancy obese are well documented [1, 6]. Excessive gestational weight gain (EGWG) as defined by the American Academy of Sciences Institute of Medicine (IOM) is also an independent predictor of adverse pregnancy and birth outcomes [6, 7]. The IOM weight gain guidelines devised in 1990 and revised in 2009 are the most widely cited guidelines for gestational weight gain [8, 9]. In the absence of Australian-based gestational weight gain guidelines, the IOM guidelines have been largely adopted as the standard reference [10, 11]. These guidelines recommend that women who are underweight at the beginning of pregnancy gain more weight than women who are overweight or obese [9].

Weight gain in excess of the IOM guidelines has been associated with both short and long term health risks, including pre-eclampsia, gestational diabetes, caesarean section, large for gestational age infants, postpartum weight retention and childhood obesity [12–14]. Evidence suggests that it is more common for women to gain weight above the IOM guidelines than within or below. In a large retrospective cohort study in the United States (*n* = 20,456), Stotland et al. [14] observed that more women gained above the IOM guidelines (43%) compared to those that gained within (37%) or below (20%). An Australian prospective cohort study of pregnancy weight gain (*n* = 664) similarly found 38% of women gained in excess of the IOM weight gain ranges [15]. Fifty-six percent of women who were overweight and obese (BMI ≥25 kg/m^2^) had EGWG compared to 30% of women with a BMI <25 kg/m^2^ [15]. Furthermore, in the majority of studies included in a recent systematic review, 47–72% of obese women had EGWG according to the IOM ranges [16].

Addressing EGWG has become a public health priority. Intervention studies have primarily focused on diet and physical activity either alone or in combination [17]. The most recent Cochrane review identified 65 randomised controlled trials (RCTs) of diet and/or exercise interventions. In an analysis of 24 included trials (*n* = 7096) diet, exercise or both in combination reduced EGWG on average by 20% (average risk ratio (RR) 0.80, 95% confidence intervals (CI) 0.73 to 0.87). However no differences were observed for the adverse outcomes of pre-eclampsia, infant macrosomia (birth weight >90^th^ centile) or caesarean birth [17].

In the real world setting there are substantial barriers to upscaling diet and exercise interventions at the population level. These include limited access to specialist staff, time constraints, financial implications and motivation to engage in such interventions as part of clinical practice [18].

One gestational weight gain intervention that is feasible at a population level (i.e. low cost and easy to administer) is weighing during routine antenatal care. The schedule of antenatal care appointments consisting of 7–12 regular visits for low risk women with maternal health care providers, presents an opportunity for health promotion interventions to be trialled. The visits additionally provide a window of opportunity for potential behaviour change and lifestyle modification [19, 20]. A recent pilot study evaluating the feasibility of regular weighing in the context of routine antenatal care reported that weighing took on average 1–2 min of a midwife’s time, was simple to do, and did not significantly add to midwives existing workloads [21]. A qualitative analysis of pregnant women’s experience of routine weighing reported that weighing during antenatal appointments was an acceptable intervention that when introduced did not cause distress or anxiety [22].

The stand-alone practice of weighing in the field of weight management has been successful in aiding non-pregnant adults achieve weight loss, weight maintenance and prevent weight gain as a self-monitoring/self-regulation strategy [23–25]. However, this has not been demonstrated in pregnancy. Weighing was originally introduced during the 1940’s as a vital sign of pregnancy, considered useful for the detection of low birth weight infants and pre-eclampsia [26]. Weighing declined in practice during the 1990’s and ceased to be recommended as a sign for adverse pregnancy outcomes by the British National Institute of Health and Care Excellence (NICE) in 2003, due to a deficit in evidence that it was an effective screening tool [26–29].

The practice of weighing is limited to the first antenatal visit in Australia and the United Kingdom for the purposes of calculating an early pregnancy BMI [11, 30]. The risks and prevalence of women entering pregnancy obese and exceeding the IOM gestational weight gain guidelines have caused health care providers necessary concern and led to develop the development of antenatal care pathways, recommending a return to weighing during all antenatal care visits [6, 31].

Therefore, this systematic review aimed to summarise the body of high quality evidence and determine any effect of routine antenatal weighing as a stand-alone intervention to reduce pregnancy weight gain and, in particular, prevent EGWG.

## Methods

This review follows the Preferred Reporting Items for Systematic Reviews and Meta-Analysis (PRISMA) [32].

### Search strategy

An a priori review protocol and eligibility criteria were devised, with consideration given to the research question, study design, population, intervention and outcomes (see Additional file 1). An electronic search of seven databases was conducted, including Medline, Embase, Maternal and Infant Care (via Ovid; http://www.ovid.com/), Cumulative Index to Nursing and Allied Health Literature (CINAHL) (via EBSCO http://www.ebsco.com/cinahl), Scopus (via http://www.scopus.com), Web of science (http://apps.webofknowledge.com ) and the Cochrane library (via http://www.cochranelibrary.com).

The initial search was conducted in November 2014 with the assistance of a research librarian (DB) using the following keywords and Boolean operators: “pregnant” OR “pregnancy” AND “weight gain” OR “weighing” AND “randomised controlled trial” OR “clinical trial” OR “random*” (see Additional file 2). All searches were limited to English language and to human studies. No date limits were applied. The Cochrane Library was searched separately to identify any previously conducted systematic reviews in the area. The search was updated in January 2016 to ensure recent evidence was captured (see Additional file 3). The database search results were exported into reference management software.

### Study selection

In the first round, publication titles and abstracts were screened independently by at least two reviewers (SMF, RMT, AJH) according to inclusion and exclusion criteria outlined in Table 1. Articles not meeting the eligibility criteria were screened out in the order of (i) study design, (ii) population, (iii) intervention, and (iv) outcome. Articles that met the eligibility criteria were retrieved as full texts and further reviewed by SMF and RMT. Any disagreements in the selection of studies were discussed with consensus achieved. The reference lists of retrieved studies and relevant Cochrane systematic reviews were hand searched for any relevant article not detected by the primary electronic search strategy.

**Table 1:** Inclusion/Exclusion Criteria

Inclusion criteria	Exclusion criteria
Randomised control trials with the intervention of any weight measurement, self-recorded or recorded by any health professional	Studies published in languages other than English Studies that are not randomised control trials
Studies that included pregnant women with a singleton pregnancy, of any age, weight, body mass index, without date limits	Studies in animals Multiple pregnancies
Studies that used more than one episode of weight measurement during pregnancy	Poor methodological quality studies
Neutral or good methodological quality studies	

### Quality assessment

Articles considered eligible for inclusion were assessed for methodological quality using the Academy of Nutrition and Dietetics Quality Criteria Checklist for Primary Research [33]. Cochrane suggests, it is preferable to use simple approaches for assessing validity that can be fully reported (i.e. how each trial was rated on each criterion) [34]. Similar to the Cochrane Collaboration’s tool for assessing risk of bias in each included study, the Academy of Nutrition and Dietetics Quality Criteria Checklist for Primary Research tool requires judgement about risk of bias to be made within each domain and support for the judgement with sufficient detail for potential sources of bias [34]. Two independent reviewers (SMF, RMT) undertook the assessments with a third reviewer (AJH) mentoring the reviewers through the process.

The quality checklist for primary research includes ten ‘scientific validity’ questions; four of which must be satisfactory to gain a positive rating (Q2 - bias, Q3 – comparable groups, Q6 - intervention, Q7 - outcomes) [33]. Answers were supplied as either "YES" meeting the criteria, "No" not meeting the criteria, or “Unclear” if the criteria was not clearly described. Articles were rated as positive (+) if the validity questions 2, 3, 6, 7, and at least one additional question were answered as “YES”; negative (−) if “No” was answered for 6 or more of the validity questions; or neutral (⦸) if answers to questions 2, 3, 6, or 7 did not indicate that the study was exceptionally strong [33]. Quality assessments of included studies are presented in the results.

### Data extraction

Relevant data were extracted by two reviewers (SMF, AJH) and entered into a Microsoft Excel spreadsheet. Data included: authors, year of publication, sample size, population characteristics, intervention and duration of the study, measures of compliance and outcomes. Weight gain outcomes included: total gestational weight gain (kg), gestational weight gain by pre-pregnancy BMI (kg/wk), and EGWG according to IOM guidelines. Pregnancy, infant and birth outcomes included: infant birth weight, macrosomia (>90^th^ centile), intrauterine growth restriction (<10^th^ centile), instrumental birth, caesarean birth, combined pregnancy induced hypertension (PIH) and pre-eclampsia (PE), gestational diabetes mellitus (GDM), infant hypoglycaemia, and Apgar <7 at 5 min.

### Statistical analysis

Meta-analysis was conducted using the mean and standard deviation for continuous outcomes and counts for categorical outcomes. A fixed-effects model using inverse variance weights was conducted. Fixed-effect models weight studies according to the amount of information they contribute, whereas random-effects models incorporate an estimate of between-study variation (heterogeneity) in the weighting. The fixed-effect assumption is that the true treatment effect is the same in each study, despite any differences in study protocols [35]. We believe a fixed effect model is appropriate as larger studies should be given more weight than smaller ones, and as there are few studies used in our meta-analysis, using a random effects model would provide poor estimates of the distribution of the intervention effects.

Forest plots with unstandardised effect size are reported for continuous variables using weighted mean difference (WMD) and 95% confidence intervals. Categorical outcomes are reported as odds ratios (OR). BMI outcomes were combined across studies to form a single outcome. Test of significance were set at the *p* < 0.05 level with all statistical analyses programmed using Stata Statistical Software [36].

## Results

### Search results

**Fig. 1:**
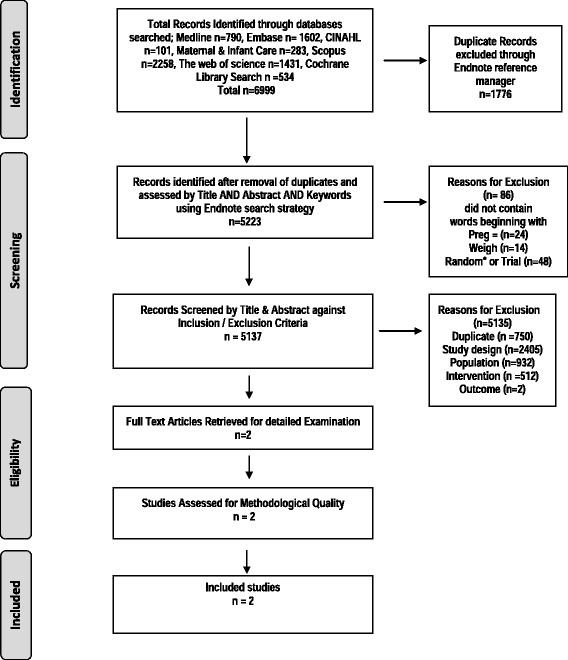
Study Selection Flowchart. Flow chart adapted from Adapted From: Moher D, Liberati A, Tetzlaff J, Altman DG, The PRISMA Group (2009). Preferred Reporting Items for Systematic Reviews and Meta-Analyses: The PRISMA Statement. PLoS Med 6 (6): e1000097. doi:10.1371/journal.pmed100

A flowchart detailing the screening and selection of studies is shown in Fig. 1. The broad search identified 6465 articles (*n* = 5223 after removal of duplicates). Initial screening of the title and abstract excluded 4067 articles. Two full text papers were then assessed and both were eligible for quality checking and meta-analysis. Hand searching did not identify any further articles for assessment.

### Study characteristics

The characteristics of studies included in this review are outlined in Table 2. Briefly, both studies were conducted in Australia. The study populations were women of any parity with singleton pregnancies enrolled during early pregnancy. Two types of weighing interventions were trialled. Jefferies et al. [37] used a self-weighing regime where women were instructed to record and document their own weight at 16, 20, 24, 28, 30, 32 and 36 week’s gestation. The control group were weighed at recruitment (≤14 week’s gestation) and at 36 week’s gestation. Both groups received standard antenatal care [37]. The second study by Brownfoot et al. [38] trialled the intervention of clinician weighing of pregnant women during scheduled antenatal care visits. The control group were weighed at the time of recruitment into the study (<21 weeks gestation) and again at 36 weeks gestation only [38]. Both groups received standard antenatal care following the participating hospitals guidelines. Both studies used an intention-to-treat analysis but had low loss to follow-up (<9%).

**Table 2:** Characteristics of included studies

Author(s), Year Study, Title, Design, & Country	Aim, quality rating	Population characteristics	Intervention, duration of study	Compliance measure(s)	Outcome(s)	Conclusions	Limitations
Jefferies, K., Shub, A., Walker, SP., Hiscock, R & Permezel, M. 2009, Reducing excessive weight gain in pregnancy: a randomised controlled trial. RCT, Melbourne, Australia.	To assess the effect of regular weight measurements and advice about the recommended (IOM 1990) weight ranges on gestational weight gain (GWG). Neutral (−)	Pregnant women recruited before ≤14 week’s gestation. Age >18 years.,<45 years., singleton pregnancy. English speaking, no pre-existing Type1 or 2 diabetes Intervention (I_1_) *n* = 148 (−23), Control (C) *n* = 138 (−27)	(I_1_) Weight measurements + advice compared to standard antenatal care (C). BMI calculated at first antenatal visit and advice on optimal weight gain given as per IOM 1990 guidelines. I_1_ self-weighing recorded on participants own antenatal card at first visit, 16, 20, 24, 28, 30, 32, 34 and 36 weeks. (C) Weighed at first visit and at 36 weeks only.	(I_1_) Weight self-recorded on personalised measurement card (tabular or graphical), using scales at hospital or participant’s home until 34 weeks (I_1_) + (C) weighed at recruitment & 36 weeks on hospital scales.	Mean difference in weight gain (Kgs/week) and between BMI subgroups. Total weight gain and proportion gaining in excess of the IOM 1990 weight gain guidelines. Maternal & neonatal pregnancy and birth complications	No difference in total weight gain Kgs/week) between (I_1)_ and (C). A statistically significant reduction in GWG (Kgs/Week.) between (I_1_) and (C) in overweight BMI subgroup only (mean difference of 0.12 kg/week (95% CI, 0.03 to 0.22), *p* = 0.01.	Weight measurements were largely self-reported based on home and hospital scales. There was no measure of participation compliance with the (I_1_). A small sample size was used with inadequate power to detect differences between groups for weight gain above IOM 1990 guidelines, pregnancy and neonatal complications.
Brownfoot, FC. Davey, MA. & Kornman, L. 2016 Routine weighing to reduce excessive antenatal weight gain: A randomised controlled trial. RCT Melbourne, Australia.	To assess the effect of clinician weighing at each antenatal visit with advice on appropriate GWG using the IOM 2009 weight gain in pregnancy guidelines. Positive (+)	Pregnant women recruited <21 weeks gestation. Age >18 years <45ys, singleton pregnancy. English speaking, no co morbidities or substance abuse identified. Intervention (I_1_) *n* = 386 (−17), Control (C) *n* = 396 (−24)	(I_1_) Weight recorded by a clinician at each antenatal appointment and documented in hospital antenatal record. The treating clinician encouraged to discuss weight gain (no scripted responses used). (C) Routine antenatal care including advice of appropriate weight gain within the IOM 2009 ranges. Both groups weighed at recruitment with BMI calculated. The (C) weighed again at ≥36 weeks gestation.	(I_1_) Weight documented in hospital antenatal records at appointments by attending clinicians. (C) Weighed at recruitment and ≥ 36 weeks only and documented on hospital antenatal record. Data collected from the antenatal hospital record, mean frequency of weight measurements reported for both groups.	Mean difference in weight gain per week (Kgs/week) and between BMI subgroups. Proportion gaining within, less than and more than the IOM 2009 weigh gain ranges. Maternal & neonatal pregnancy and birth complications	No statistically significant differences reported in mean weight gain per week (I_1_) 0.54 kg (±0.28) & (C) 0.53 kg (±0.24) *p* = 0.63 (*p* = 0.05). No difference in proportion of women gaining weight within, less than or more than IOM 2009 guidelines. No differences between groups for all neonatal and maternal complications.	Study not powered to detect a between group differences for all maternal and neonatal pregnancy and birth complications reported.

### Study quality

A summary of the quality assessment is presented below in Table 3.

**Table 3:** Summary of the quality assessment for the included studies

First author, year of publication (reference)	Jefferies et al. 2009 [37]	Brownfoot et al. 2016 [38]
Validity questions		
1. Was the research question clearly stated?	Y	Y
2. Was the selection of study subjects/patients free from bias?	Y	Y
3. Were study groups comparable?	Y	Y
4. Was method of handling withdrawals described?	Y	Y
5. Was blinding used to prevent introduction of bias?	Y	Y
6. Were intervention/exposure factor or procedure and any comparison(s) described in detail?	N	Y
7. Were outcomes clearly defined and the measurements valid and reliable?	Y	Y
8. Was the statistical analysis appropriate for the study design and type of outcome indicators?	Y	Y
9. Were conclusions supported by results with biases and limitations taken into consideration?	Y	Y
10. Is bias due to study’s funding or sponsorship unlikely?	Y	Y
Overall quality	N	P

American Dietetic Association Quality Criteria Checklist for Primary Research, *Y* yes, *N* no,

*P*, positive rating; *N* neural rating

Both studies answered “Yes” to all relevance questions. Of the four validity questions, the study by Jefferies et al. [37] received a “NO” for question 6, with reviewers questioning participant compliance with the intervention and validity of instruments within the intervention group. The corresponding author of the paper was contacted seeking additional information and clarification, however, no further information could be provided. This paper received a neutral quality rating with a score of 9 out of a possible 10 [18].

The second study conducted by Brownfoot et al. [38] reported sufficient information within their publication receiving a “YES” for all scientific validity questions. The paper gained a total score of 10 and received a positive quality rating.

### Analysis results

**Fig. 2:**
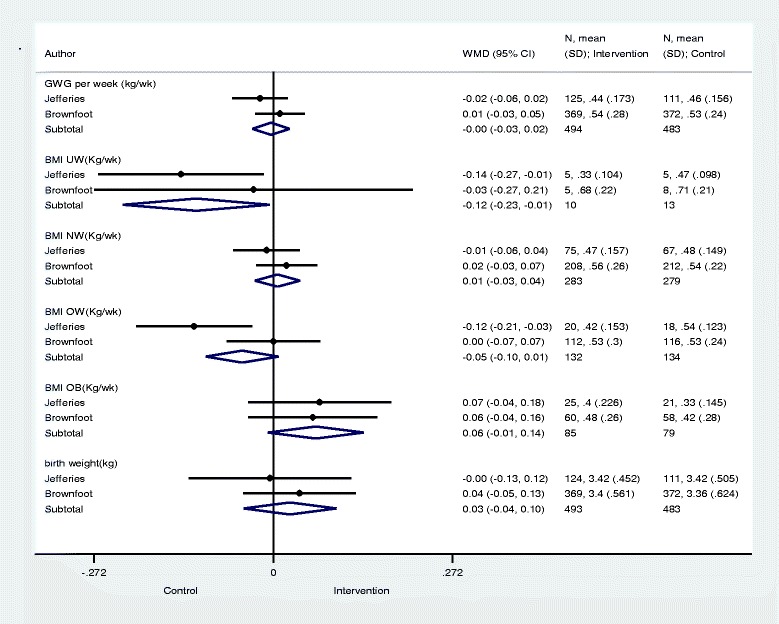
Results for Continious Variables and Tests of Significance. Significance test(s) of Weighted Mean Difference (WMD = 0), Gestational Weight Gain (GWG) per week (kg/wk) z = 0.23, *p* = 0.815; Body Mass Index (BMI) Underweight (UW) (Kg/wk) z = 2.06, *p* = 0.040; BMI Normal Weight (NW) (Kg/wk) z = 0.36, *p* = 0.716; BMI Overweight (OW) (Kg/wk) z = 1.68, *p* = 0.094; BMI Obese (OB) (Kg/wk) z = 1.74, *p* = 0.081; Birth Weight (kg) z = 0.70, *p* = 0.481

Meta-analysis of continuous outcomes is displayed in Fig. 2. There was no difference in total gestational weight gain between the intervention (*n* = 494) and control groups (*n* = 483). In the sub-group analysis of weight gain by BMI category a statistically significant difference was found for underweight women. The amount of weight gained in underweight women was 0.12 kg/week (*n* = 23, *p* = 0.040) less in the intervention group compared to control.

**Fig. 3:**
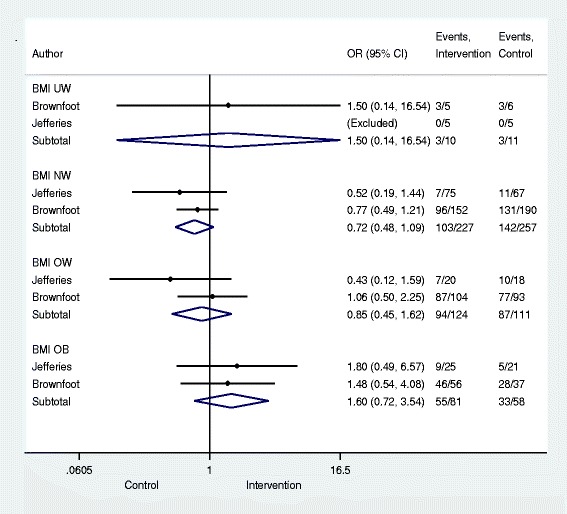
Proportion of weight gain exceeding the IOM ranges and tests of significance. Significance test(s) of Odds Ratio (OR) =1; Body Mass Index (BMI) Underweight (UW) z = 0.33, *p* = 0.741; BMI Normal Weight (NW) z = 1.55, *p* = 0.122; BMI Over Weight (OW) z = 0.50, *p* = 0.617; BMI Obese (OB) z = 1.15, *p* = 0.250

There were no differences in the total proportion of women exceeding the IOM weight gain ranges between intervention (*n* = 290) and control (*n* = 230): OR 1.10 (95%CI, 0.81 to 1.50). Data on EGWG by BMI category are presented in Fig. 3 and show no differences in the intervention and control groups.

**Fig. 4:**
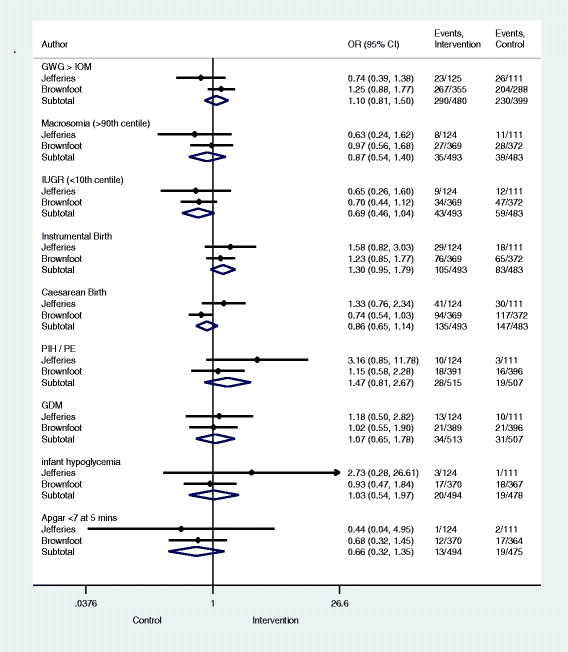
Pregnancy and Birth Outcomes and Tests of Significance. Significance test(s) of Odds Ratio (OR) =1; Gestational Weight Gain (GWG) > Institute of Medicine (IOM) z = 0.63, *p* = 0.532; Macrosomia (>90th centile) z = 0.58, *p* = 0.560; Intra Uterine Growth Restriction (IUGR) (<10th centile) z = 1.76, *p* = 0.079; Instrumental Birth z = 1.62, *p* = 0.105; Caesarean Birth z = 1.06, *p* = 0.288; Pregnancy Induced Hypertension (PIH)/Pre-Eclampsia (PE) z = 1.26, *p* = 0.206; Gestational Diabetes Mellitus (GDM) z = 0.27, *p* = 0.787; Infant hypoglycemia z = 0.10, *p* = 0.917; Apgar <7 at 5 mins z = 1.15, *p* = 0.252

For all secondary pregnancy and birth outcomes (including birth weight on Fig. 2) no significant differences were found between intervention and control as per Fig. 4.

We performed a post-hoc power calculation to determine the minimum detectable difference in total gestational weight gain for the pooled total of 977 participants, distributed approximately evenly between intervention and control groups. The minimum detectable difference was approximately 735 g in total gestational weight gain (~20 g per week), with 80% power, α = 0.05, and SD ± 4.1 kg [37].

## Discussion

This systematic review of RCTs aimed to determine the evidence base for weighing as a stand-alone intervention to reduce pregnancy weight gain and prevent EGWG. Two RCTs were retrieved and meta-analysed. Together they suggest that weighing, as a stand-alone intervention during routine antenatal care, is no better at reducing total pregnancy weight gain or preventing weight gain in excess of the IOM weight gain ranges than routine antenatal care.

A statistically significant lower rate of gain (kg/wk) was observed in women in the underweight BMI category between intervention and control. This finding should be interpreted with caution as it was derived from a BMI group that only included 23 women and due to multiple comparisons across BMI sub-groups could be due to random chance alone. However, it is also plausible that underweight women may be more sensitive to weighing and this practice may have an impact on their rate of weight gain._._ Nohr et al. [39], in a large Danish birth cohort study (*n* = 60,892), determined that women who were categorised as underweight at the beginning of pregnancy (BMI <18.5 kg/m2) who had lower rates of GWG (<10kgs) were found to be more at risk of giving birth to small for gestational age infants (OR 1.9, 95%CI 1.7 to 2.1) [39]. Based on the existing evidence the IOM in 2009 recommended that underweight women should gain towards the upper limits of the weight gain ranges specifically to prevent small for gestational age infants [40].

It is extremely interesting that only two recent trials contributed data for this review, given the increased prevalence of obesity and EGWG and changes in practice over time. Additionally, weight gain is characteristic of pregnancy progression and a well-recognised determinant of fetal growth. There is convincing evidence that GWG is associated with infant birth weight: lower GWG is associated with low birth weight and greater GWG is associated with large for gestational age infants [12].

In light of this evidence it is difficult to reasonably explain why antenatal guidelines restrict the practice of routine antenatal weighing and not consider it as an important predictor of pregnancy outcomes, similar to serial measures of blood pressure.

Restricting routine weighing is in direct contrast to the IOM (2009) weight gain guidelines that specifically advise for pregnant women to be weighed at the initial and all subsequent antenatal visits to detect abnormal patterns of pregnancy weight gain [9]. The guidelines recommend that health care providers work in partnership with women to set individual weight gain targets according to their BMI and for weight gains to be graphically documented to enable women to be aware of their weight gains and educate them on the importance of appropriate pregnancy weight gain [9].

Dimperio et al. [41] in response to recommendations that routine weighing should be abandoned, argued that weighing was more than just a stand-alone pregnancy intervention and rather presented health care practitioners with the opportunity to counsel women before weight gains became extreme, advocating that weighing is a valuable screening tool rather than a diagnostic tool for adverse pregnancy outcomes [41].

Weighing as a stand-alone intervention may not be effective for reducing pregnancy weight gain and EGWG under controlled conditions however given the prevalence and risks associated with weight gains outside of the IOM guidelines it is negligent of maternity care providers not to address weight gain in pregnancy. Maternity care providers need to be working in partnership with women to achieve the IOM weight gain in pregnancy targets, monitoring their progress and providing feedback on that progress. Therefore, we recommend further research be undertaken into the impacts and acceptability of this intervention within various health care settings and models of pregnancy care, using both experimental and qualitative research methods.

### Strengths

We have conducted a methodically rigorous and contemporary search to determine if weighing as a stand-alone intervention can reduce EGWG. All available experimental evidence has been assessed and reported in accordance with the PRISMA guidelines [32] and an appropriate methodological quality checklist [33].

### Limitations

Although the included RCTs were deemed good quality, with neutral and positive quality ratings, the following limitations need to be considered. Giving benefit of the doubt, blinding within both studies was rated as adequate, even though neither the participant nor clinicians/researchers (who were also the outcome assessors) were blinded to the intervention. This is because the quality check question is phrased with the qualifier “as appropriate”. Jefferies et al. [37] reported that participants were blinded to the purpose of the study, however, discussed that researchers conducting the study were not blinded to treatment groups. No participant blinding was used in the study by Brownfoot et al. [38] because of the nature of the intervention, and this was acknowledged in their limitations. Reviewers gave consideration to each study’s methods and concluded that true blinding would be extremely difficult.

Secondary outcomes within both studies including, proportion of women gaining weight above the IOM recommendations, pregnancy birth and neonatal outcomes were not pre specified within each study’s statistical analysis plan. These outcomes were not adequately powered to detect a difference between intervention and control limiting the generalisability of these findings.

The decision to exclude studies published in a language other than English was made a priori, for pragmatic reasons. Authors acknowledge that there is potential for this exclusion to have contributed to the low number of included studies.

## Conclusion

This systematic review and meta-analysis concludes that weighing, as a stand-alone intervention is neither worse nor better at reducing excessive gestational weight gain than routine antenatal care alone. In light of the presented evidence we recommend that where antenatal guidelines advise women to gain weight within the IOM weight gain ranges that they be enacted in their entirety recommending that women be weighed at the first and all subsequent antenatal visits. We additionally recommend that further research studies be conducted to assess the impact and acceptability of weighing in pregnancy.

## Additional files

Additional file 1. Study Protocol. Description of data: Systematic review study protocol. (DOCX 17 kb)
Additional file 2. Primary Search Strategy November 2014. Description of data: This file contains all details of the primary systematic review search strategy conducted in November 2014. Including databases, search terms and number of citations retrieved. (DOCX 18 kb)
Additional file 3. Primary Search Strategy (Updated January 2016). Description of data: This file contains all details of the updated primary systematic review search strategy conducted in January 2016. Including databases, search terms and number of citations retrieved. (DOCX 18 kb)
Additional file 4. Spreadsheet of values used for data analysis. (XLSX 13 kb)

## Abbreviations

BMI: Body mass index
CI: Confidence interval
EGWG: Excessive gestational weight gain
GDM: Gestational diabetes mellitus
IOM: Institute of Medicine
NICE: National Institute of Health and Care Excellence
OR: Odds ratio
PE: Pre-eclampsia
PIH: Pregnancy induced hypertension
PRISMA: Preferred reporting items for systematic reviews and meta – analysis
RCTs: Randomised controlled trials
WMD: Weighted mean difference
