# Supplementary material for: Weighing as a stand-alone intervention does not reduce excessive gestational weight gain compared to routine antenatal care: a systematic review and meta-analysis of randomised controlled trials
Source: BMC Pregnancy Childbirth. 2017 Jan 17;17:36. doi: 10.1186/s12884-016-1207-2 (PMC5240423; doi:10.1186/s12884-016-1207-2)
Supplement: Additional file 1. — Study Protocol. Description of data: Systematic review study protocol. (DOCX 17 kb) [file 12884_2016_1207_MOESM1_ESM.docx]

Supplementary file 1. Study Protocol

**Does routine weighing during pregnancy reduce excessive gestational weight gain?**

**A systematic review and meta-analysis of randomised controlled trials: Protocol**

**Research Question:**

Does routine weighing during pregnancy reduce excessive gestational weight gain?

*P (population) pregnant women, any age*

*I (intervention) weight measurement during routine antenatal care (exclusive of other dietary and lifestyle interventions)*

*C (comparator) control group*

*O (outcomes) reduction in weight gain*

**Study design:**

A systematic review and meta-analysis of randomised controlled trials

**Interventions:**

Any intervention providing regular weighing to pregnant women, exclusive of other dietary and lifestyle modification as a means of avoiding excess weight gain/reducing weight gain

**Outcome variables**

***Primary***

- Weight gain / weight change (total weight gains, weight gains within, below and above IOM ranges)

***Secondary***

- Pregnancy outcomes (gestational diabetes, hypertension, pre-eclampsia)
- Birth Outcomes (caesarean birth, instrumental birth)
- Infant outcomes (infant birth weight, Apgar score, intrauterine growth restriction, macrosomia)

**Inclusion criteria**

- Pregnant women *(any age)*
- Singleton pregnancy
- More than one measure of weight during pregnancy
- Randomised controlled trials
- Neutral and good methodological quality studies

**Exclusion criteria**

- Studies not published in English
- Animal studies
- All other study designs, except randomised controlled trials
- Poor methodological quality studies

**Subgroups / sensitivity analysis**

- BMI sub-groups (underweight, normal weight, overweight and obese)
- Diabetes or other medical conditions
- Country’s income level (OECD classification)
- Number of antenatal visits
- Care provider (midwife, GP, obstetrician etc.)

**Databases to search:**

- MEDLINE
- CINAHL
- Embase
- Maternal & Infant Care
- Scopus
- The Web of Science

**Search terms / keywords**

- pregnant
- pregnancy
- weight gain
- weighing
- randomised controlled trial
- clinical trials

**Data extraction**

- First Author, Year, Country and Reference Number
- Study design
- Number of participants
- Participant characteristics
- Intervention *(who gives the intervention i.e. self reported, how long the intervention is given for; how the intervention is delivered; marker of compliance)*
- Maternal age, country, any demographic factors
- Study aim
- Statistical analysis
- Conclusion
- Limitations
- Methodological quality
