# Supplementary material for: Weighing as a stand-alone intervention does not reduce excessive gestational weight gain compared to routine antenatal care: a systematic review and meta-analysis of randomised controlled trials
Source: BMC Pregnancy Childbirth. 2017 Jan 17;17:36. doi: 10.1186/s12884-016-1207-2 (PMC5240423; doi:10.1186/s12884-016-1207-2)
Supplement: Additional file 3. — Primary Search Strategy (Updated January 2016). Description of data: This file contains all details of the updated primary systematic review search strategy conducted in January 2016. Including databases, search terms and number of citations retrieved. (DOCX 18 kb) [file 12884_2016_1207_MOESM3_ESM.docx]

Supplementary File 3.

Primary Search Strategy (Updated January 2016)

| DATABASE: | Medline  (mp) | Embase Classic + Embase (mp) | Maternal & Infant Care  (mp) | CINAHL |
| --- | --- | --- | --- | --- |
| DATE: (dd/mm/yy) | 25/ 01/ 16 | 25/ 01/ 16 | 25/ 01/ 16 | 25/ 01/ 16 |
|  |  |  |  |  |
| KEYWORD SEARCH TERMS |  |  |  |  |
| 1. pregnancy | 799584 | 826055 | 86091 | 152466 |
| 2. pregnant | 130794 | 202279 | 26554 | 21830 |
| 3. 1 or 2 | 811763 | 873467 | 90589 | 155224 |
| 4. weight gain | 53411 | 101332 | 3102 | 12518 |
| 5. weighing | 19582 | 37291 | 1490 | 2831 |
| 6. 4 or 5 | 72394 | 133212 | 4472 | 15247 |
| 7. randomi?ed control* trial | 414033 | 496392 | 3591 | 83324 |
| 8. clinical trial | 597309 | 1087271 | 1402 | 157588 |
| 9. random* .ti,ab. | 710244 | 1059961 | 17080 | 44 |
| 10. 7 or 8 or 9 | 1148653 | 1870256 | 17409 | 216382 |
| 11. 3 and 6 and 10 | 1027 | 1924 | 259 | 221 |
| 12. limit 11 to English & Human | 714 | 1507 | 259 | 159 |
| 13. limited date 2014 to 2016 or current | 714 | 1507 | 259 | 159 |
| Total retrieved: | 115 | 333 | 55 | 39 |
| [mp=title, abstract, original title, name of substance word, subject heading word, keyword heading word, protocol supplementary concept word, rare disease supplementary concept word, unique identifier]  [ti =title, ab=abstract]  **[**Medline covers the vast majority of journal articles found in PubMed, by including citations from more than 5,600 scholarly journals published around the world. The only content covered by PubMed and not Medline are manuscripts deposited in PMC (commonly would be in both) and NCBI Bookshelf (i.e. books and other documents). The advantage of searching Medline over PubMed is the ability to conduct the search using consistent Medical Subject Headings (MeSH)]. | | | | |

| DATA BASE: | Scopus | Web of Science |
| --- | --- | --- |
| DATE: (dd/mm/yy) | 25/ 01/ 16 | 25/ 01/ 16 |
| BASIC SEARCH: (per data base) | Title / Abstract / Keyword | Topic |
|  |  |  |
| KEY WORD SEARCH TERMS |  |  |
| 1. pregnancy or pregnant | 957880 | 368937 |
| 2. weight gain or weighing | 183402 | 139892 |
| 3. Randomi?ed control* trial or clinical trial or random* | 2 503177  (random* Title / Abs only) | 1577431 |
| 4. 1 and 2 and 3 | 2396 | 1372 |
| 5. Limits:  (English Language) | 2277 | 1321 |
| 6. Excluded:  *Subject area (Veterinarian) | 2169 | 1260 |
| 7. Limited date 2014 to 2016 or current | 355 | 323 |
| Total Retrieved: | 355 | 323 |
